# IRIS: Infection with RespIratory Syncytial Virus in infants—a prospective observational cohort study

**DOI:** 10.1186/s12890-022-01842-1

**Published:** 2022-03-15

**Authors:** Martin Wetzke, Dominik Funken, Mathias Lange, Levente Bejo, Sibylle Haid, Joao G. Tereno Monteiro, Katharina Schütz, Christine Happle, Thomas F. Schulz, Jürgen Seidenberg, Thomas Pietschmann, Gesine Hansen

**Affiliations:** 1 Department of Pediatric Pneumology, Allergology and Neonatology, Hannover Medical School, Hanover, Germany; 2 German Center for Infection Research (DZIF), Site Hanover-Brunswick, Germany; 3 Biomedical Research in End-Stage and Obstructive Lung Disease Hannover (BREATH), Hannover, Germany; 4 Department of Pediatric Pneumology and Allergology, Universitätsklinik für Kinder- und Jugendmedizin Oldenburg, Oldenburg, Germany; 5 Helios Childrens Hospital Hildesheim, Hildesheim, Germany; 6 Institute for Experimental Virology, TWINCORE Centre for Experimental and Clinical Infection Research, Hannover, Germany; 7 Cluster of Excellence RESIST (EXC 2155), Hannover Medical School, Carl-Neuberg-Straße 1, 30625 Hannover, Germany; 8 Institute of Virology, Hannover Medical School, 30625 Hannover, Germany

**Keywords:** Respiratory syncytial virus (RSV), Infants, Toddlers, Bronchiolitis, Infection, Cohort study, Genetic susceptibility

## Abstract

**Background:**

Respiratory syncytial virus (RSV) is the most common cause of acute lower respiratory tract infection in infants. Globally, RSV is responsible for approximately 3.2 million hospital admissions and about 60,000 in-hospital deaths per year.

**Methods:**

Infection with RespIratory Syncytial Virus (IRIS) is an observational, multi-centre study enrolling infants with severe RSV infection and healthy controls. Inclusion criteria are age between 0 and 36 months and hospitalisation due to RSV infection at three German sites. Exclusion criteria are premature birth, congenital or acquired bronchopulmonary or cardiac diseases, and immunodeficiency. Healthy control probands are enrolled via recruitment of patients undergoing routine surgical procedures. Blood and respiratory specimens are collected upon admission, and RSV and other pathogens are analysed by multiplex polymerase chain reaction. Different biomaterials, including plasma, nasal lining fluid, blood cells, DNA, and RNA specimens, are sampled in a dedicated biobank. Detailed information on demographic characteristics and medical history is recorded, and comprehensive clinical data, including vital signs, medication, and interventions.

**Discussion:**

The IRIS study aims to discover host and viral factors controlling RSV disease courses in infants. The approach including multi-omics characterisation in clinically well-characterized children with RSV bronchiolitis seeks to improve our understanding of the immune response against this virus. It may disclose novel diagnostic and treatment approaches for respiratory infections in infants.

*Trial registration*: ClinicalTrials.gov, NCT04925310. Registered 01 October 2021—Retrospectively registered. https://clinicaltrials.gov/ct2/show/NCT04925310?cond=NCT04925310&draw=2&rank=1

## Background

Acute lower respiratory tract infections (ALRI) are among the leading causes of childhood morbidity and mortality worldwide [1]. Respiratory syncytial virus (RSV) is the most common cause of ALRI in infants and one of the most frequent causes of hospitalisation in the first year of life [2–4]. Globally, RSV is responsible for approximately 33 million episodes of ALRI in children younger than 5 years, leading to 3.2 million hospital admissions and 59,600 in-hospital deaths [2]. RSV of the family *Pneumoviridae* is a single-stranded RNA-virus with two co-circulating antigenic subtypes (RSV A and B). Human-to-human transmission occurs via droplets and causes seasonal infections in all age groups [5].

Almost every child encounters RSV until the age of 2 years. Approximately 45% of RSV-associated hospitalisations and in-hospital deaths occur in children under 6 months [2]. Premature birth, bronchopulmonary dysplasia, and cardiac defects predispose infants to severe RSV disease courses, but most severe RSV infections occur in otherwise healthy children with thus far unknown risk factors [4]. Genetic factors appear to play a central role in RSV susceptibility [6]. For example, the rate of RSV-associated hospitalisation in children of native US-American origin is three times higher than the general US population [7]. Twin studies from Denmark demonstrated a significantly higher concordance rate in susceptibility to severe RSV infection in identical twins than fraternal twins [8, 9]. Several studies have reported on the role of genes regulating the innate immune and variations in alveolar surfactant protein genes in modulating RSV immunity [6, 10, 11]. However, informative genetic markers predicting RSV disease risk in infants are unavailable. Severe RSV-induced disease in children is believed to occur either due to uncontrolled viral replication or excessive immune response, or both [12]. It has also been proposed that different RSV subtypes may cause distinct clinical outcomes [13, 14]. Several immune cells, such as neutrophils and cytotoxic T cells, are relevant in RSV immunity [15, 16]. Also, B cells were shown to play an essential role in the immune response against RSV, especially in severe and fatal cases [17, 18]. Accordingly, different immunological factors and inflammatory and regulatory cytokine expression patterns have been associated with severe RSV courses [12, 19]. However, the complex interplay of innate and adaptive immune responses in severe RSV is still poorly understood. Rapid identification of infants at high risk for severe RSV infection and effective prophylactic measures could profoundly affect global infant morbidity and mortality [3]. Thus far, despite considerable efforts for over 5 decades, neither effective antivirals nor active vaccines are available [3]. Prophylactic use of the monoclonal anti-RSV antibody palivizumab in children at high risk (preterm, cardiac defects) was shown to decrease hospitalisation rates [20, 21]. We initiated the Infection with RespIratory Syncytial Virus (IRIS) study to improve our understanding of the RSV immune response in infancy. This prospective multicenter study aims at deciphering host and viral factors regulating RSV severity in infants. The study evaluates genetic risk factors by employing comprehensive immune phenotyping, unbiased genome-wide sequencing, and multi-omics techniques. It assesses the potential of existing and new diagnostic and treatment strategies in severe RSV infection.

## Methods/design

### Aims

IRIS aims to understand susceptibility factors for severe RSV infection and the immune response against RSV in infants. The study will provide current and in-depth clinical and molecular data on RSV immunity.

The specific aims of IRIS are:

- to identify host susceptibility factors for severe RSV disease courses in infancy,
- to identify viral risk factors for severe RSV infections in infancy,
- to identify and validate novel biological markers identifying children at risk for severe RSV infections,
- to develop laboratory and clinical scores for early risk stratification,
- to analyse RSV strains and coinfections in children with RSV via comprehensive virome screenings,
- to validate the functional role of newly identified susceptibility and risk factors in vitro and in vivo
- to participate in decision-making processes for guidelines and prevention strategies to ultimately improve care and prevention of RSV during infancy in the future.

### Sample selection

The IRIS study is designed as a multicentric, prospective, observational study initiated at Hannover Medical School, Germany.

The study enrols hospitalised children with confirmed RSV infection between the first month and the second year of life. The diagnosis of RSV is evaluated by point-of-care testing (POCT, Sofia, Quidel, Kornwestheim, Germany), and positive findings are confirmed by polymerase chain reaction (PCR). Exclusion criteria are premature birth, congenital or acquired bronchopulmonary or cardiac diseases, and immunodeficiency. Healthy control probands are enrolled via the recruitment of patients undergoing routine surgical procedures. Written informed consent is obtained from all parents and caregivers.

### Study centres

Three local study centres in northern Germany (Hannover Medical School, Children’s University Hospital Oldenburg, and Helios Children’s Hospital Hildesheim) contribute. All study sites are tertiary care hospitals and underwent extensive training in recruitment, biosample acquisition and processing, data collection and entry, logistics, and security.

### In- and exclusion criteria

The study enrols hospitalised children with confirmed RSV infection between the first month and the second year of life. The diagnosis of RSV is evaluated by point-of-care testing (POCT, Sofia, Quidel, Kornwestheim, Germany), and positive findings are confirmed by polymerase chain reaction (PCR). Exclusion criteria are premature birth, congenital or acquired bronchopulmonary or cardiac diseases, and immunodeficiency. Healthy control probands are enrolled via recruitment of patients undergoing routine surgical procedures. Written informed consent is obtained from all parents and caregivers.

### Data and biomaterial collection

We will collect data at two-time points: baseline (date of inclusion in the study) and at discharge from hospital care. Upon enrollment, detailed demographic background, case history, clinical presentation, physical examination, diagnostic findings, treatment, and other patient-related items are collected (Table 1). Data on disease course, treatment, and complications are gathered. Blood and respiratory specimens are collected upon admission, and RSV and other pathogens are analysed by multiplex polymerase chain reaction (PCR). Different biomaterials, including plasma, nasal lining fluid, blood cells, DNA, and RNA specimens, are sampled in a dedicated biobank (Table 2, Hannover Unified Biobank).

**Table 1:** Patient information and clinical data collected in IRIS

Type of data	Collected variables
General information	Patient and cent identifier, date of inclusion, demographic data (e.g. date of birth, sex, region of origin, parental ethnicity), weight, height
Case history	Date of admission, preexisting and concomitant diseases, medication prior to hospitalisation, or surgical interventions
Clinical data	Vital signs, oxygen saturation breathing ambient air, respiratory signs and symptoms, initiated treatment, complications, duration of hypoxemia, respiratory support, days of hospitalisation
Diagnostics	RSV POCT/PCR results. Optional: diff blood count, CRP, creatinin, Na, Urea, blood gas, viral pathogen screening of airway swab
Imaging	Optional: chest radiograph or ultrasound
Other data	Atopy, vitamin D supplementation, family history of airway diseases, parental diseases

**Table 2:** Biosampling and laboratory analyses in IRIS

Source	Specimen	Direct analysis	Biobank sampling; planned analyses
Upper airway tract	Nasopharyngeal aspirate or swab	PCR pathogen screening, microbiome analysis, culture (optional)	Yes; sequencing and cultures of clinical viral strains
Upper airway tract	Nasal fluid		Yes; cytokine analyses, transcriptome, microRNA analysis
Blood	EDTA	Optional: differential blood count	Yes; flow cytometry, chip-cytometry, genomics, epigenomics
Blood	Serum	Optional: CRP, creatinin, sodium, urea, blood gas	Yes; proteomics, metabolome, antibody-screening
Blood	Plasma		Yes; microRNA, metabolome

All probands receive a specific pseudonym upon enrollment, and data management and all further analyses are based on this pseudonym. To ensure that data from the study are not linked to personal identities but that proband specimens and information can be destroyed upon request, an external data trustee will manage a list relating pseudonyms to patient identities. The electronic study database is encrypted, password-protected, and de-identified (MS Access). The biobank system Centraxx (Kairos, Version: 3.18.1.18) stores logistic information on de-identified biological specimens.

### Clinical variables to be recorded

At baseline, data including patient demographics, clinical characteristics of the disease at inclusion, investigations (laboratory findings, radiology), and treatments will be obtained as personal and a case history (Table 1).

At discharge, data will include the child’s outcome, highest mode of clinical care (regular or intensive care), highest mode and duration of respiratory support and treatment, as well as complications (Table 1).

### Sample size calculation

The study recruits a convenience sample cohort; as such, the sample size is not calculated.

### Outcome

*Primary Outcome Measures* To report the number of severe RSV infections in children under 3 years of age in three tertiary care centres in northern Germany.

*Secondary Outcome Measures*

- To report outcome measures of severe RSV infection under 3 years of age in children under 3 years (composite endpoint).
- To collect biomaterials from children with severe RSV infection for downstream extensive immunological characterisation.

### Data analysis and statistical plan

Demographics and analysis of clinical characteristics will be performed by applying descriptive statistics. This will include median age, co-morbidities and presenting features of the disease; moreover, to assess severity saturation levels, blood gas composition, highest mode of clinical care and respiratory support and length of stay will be analysed.

Data acquired from investigation for various biomaterials will be analysed according to statistical analysis plans before complete data analysis. This will include multivariate logistic regression analysis to examine biological risk markers for severe RSV courses.

### Ethics

Local ethic authorities have approved the study (MHH#6309_10/31/2012; Hannover Medical School).

### Infrastructure

The IRIS cohort is embedded into a well-established interdisciplinary study infrastructure at Hannover Medical School and associated partners. All necessary protocols and infrastructure were developed within the first year of funding, including an electronic information platform and biobanking procedures. These structures enable harmonised multicentric data and biospecimen collection and storage of information and samples in a central IRIS biobank hub, allowing for systematic multi-omics analyses. The biobank is embedded into the Hannover Unified Biobank (Hannover Medical School, Hannover, Germany). The reference laboratory for pathogen sequencing is located at the virology department of Hannover Medical School, Hannover, Germany.

### Data input, storage and management

Pseudonymised patients will be entered into a password secured database (Microsoft Access). Local password-secured copies of the database during recruitment in each recruiting hospital are run. These local copies are merged into one overall record at Hannover Medical School at the end of each RSV season. The database provides a comprehensive log/audit feature to record data input and changes based on user and time/date.

The biobank system Centraxx (Kairos, Version: 3.18.1.18) stores logistic information on de-identified biological specimens. Centraxx is certified according to DIN EN ISO 13485 to develop medical products.

### Biosampling and biobanking

All collected biosamples are used immediately in diagnostic procedures or processed and stored to the central biobank for regular shipment. All steps are performed according to established standard operating procedures, available to all study sites in written form, and trained before first patient inclusion. For all probands, genetic material is collected by blood sampling or collection of DNA material through buccal swapping. In those children who undergo blood sampling for diagnostic purposes, further comprehensive biosampling is conducted, including collecting serum, plasma, whole blood, and DNA and RNA isolation. Furthermore, nasal fluid and airway swabs are sampled (Table 2). Airway specimens are analysed by PCR and cultured to characterise RSV strains and screened for possible coinfections (e.g., viral pathogens, current screening includes adeno-, corona-, influenza A and B, human metapneumo-, parainfluenza 1–4, and rhinovirus). Further planned analyses include screening for microRNAs in plasma and nasal fluid and multiplex analyses of cytokines and metabolic parameters (Table 2).

### Research dissemination and patient involvement

Gathered data will be published in scientific articles, preferably in open-access journals, and presented at conferences. If eligible, citations to repositories that host the underlying data will be included, together with details of any software used to process it. The output management plan will be reviewed and harmonised in regular meetings throughout the project. IRIS researchers will be open to collaborations upon reasonable request at any time point. Furthermore, the IRIS network will participate in decision-making processes for guidelines and prevention strategies to ultimately improve care and prevention of RSV during infancy in the future. Probands guardians can choose whether a comprehensive summary shall be provided to them after completing the study. We aim to actively involve patient families in the future development of the study design.

## Discussion

Regardless of economic standard, RSV is a significant healthcare burden associated with high global infant morbidity and mortality [2]. Despite decades of efforts in this field, no effective broad prevention or treatment strategies have existed until now [3]. The IRIS study network aims to improve this situation by delivering comprehensive clinical and multi-omics data on severe RSV disease courses during infancy. With our approach, we want to improve our knowledge of the genetic and immunological background of RSV immunity and improve the management of severe RSV infections in small children. Multi-omics data including whole-exome sequencing, expression of immunological factors on RNA-, microRNA-, protein-level, and pathogen spectra will be gathered. The collected comprehensive demographic and clinical data will be used for complex biostatistical analyses to assess novel risk stratification scores and biomarkers to identify children at particular risk for severe RSV infections. Thus far, more than 200 children were included in the IRIS cohort at three study sites, with ongoing recruitment and a growing study network. Initial analyses of samples from the pilot phase addressed genetic polymorphisms in interferon (IFN) regulated genes [10, 22] and identified new genetic risk factors for severe RSV [23]. Besides the analysis of IFN stimulated genes, whole-exome sequencing will next focus on genetic variants modulating alveolar surfactant and mucin expression and innate immunity [12]. We plan to screen airway and blood samples to express soluble and cellular markers by multi-omics approaches and flow and chip cytometry to assess immune signatures characteristic for severe RSV disease courses. Several factors such as immunoglobulins, B cell-activating factor (BAFF), C-X-C motif chemokine ligand 10 (CXCL10), type I IFN, mucins, and others were shown to be differentially regulated in the airway immune response against RSV [12, 19, 24]. Airway infiltration of T cells, neutrophils, and specific B cell subsets have been shown to occur during RSV bronchiolitis [16–18]. Accordingly, airway and blood specimens of IRIS probands will be screened for such markers, and distribution and activation of immune cells will be assessed and compared to respective patterns in healthy controls. Different infection courses also result in distinct levels of plasma markers of oxidative stress [25]; the metabolome of IRIS probands will be analysed by mass spectrometry. Moreover, previous studies suggest that specific co-infections correlate with severe RSV courses. Therefore, the co-infection status of patients will be determined using multiplex PCR assays [26]. Genotyping of clinically isolated RSV strains will be performed to investigate RSV strain-specific effects. Particular strains will be cultured to compare their pathogenicity to standard laboratory RSV strains and other recent primary clinical RSV isolates. Methods for experimental RSV infections have been adapted and optimised for these purposes [27], and these techniques will be used for viral mapping domains responsible for antibody resistance and drug discovery [28]. These molecular in vitro studies are complemented by analyses in mouse models of RSV infection using clinical virus isolates in knockout and reporter mice to validate potential hits and gather further mechanistic insights. To achieve up-to-date and comprehensive biostatistical analysis of the multi-omics data and include novel advances in data sciences, a joint research consortium INDIRA (INtegrative Data analytIcs for Respiratory syncytiAl virus RIsk Assessment) was founded, connecting IRIS researchers to bioinformatical experts. Predictive and diagnostics tools from the cohort data will be developed using computer models and bioinformatics.

Updating and expanding the knowledge on RSV immunity in infancy will have relevant implications in future management (e.g. identifying children at high risk for severe RSV disease courses) and prevention (e.g. vaccination) of RSV. Our approach may disclose novel diagnostic and treatment strategies for respiratory infections in infants and beyond.

## Data Availability

A formal application can request data and biomaterials of the IRIS cohort. Requests should be directed to the corresponding author.

## Abbreviations

CRP: C-reactive protein
INDIRA: INtegrative Data analytIcs for Respiratory syncytiAl virus RIsk Assessment
IRIS: Infection with respiratory syncytial virus
RSV: Respiratory syncytial virus
RV: Rhinovirus
PCR: Polymerase chain reaction
